# Investigation of ceftazidime-avibactam susceptibility in clinical isolates of gram-negative bacteria

**DOI:** 10.55730/1300-0144.5530

**Published:** 2022-10-09

**Authors:** Rıdvan DUMLU, Neval YURTTUTAN UYAR, Meltem AYAŞ, Nilay AKSOY, Nur ÖZTÜRK, Ayşe Sesin KOCAGÖZ

**Affiliations:** 1 Department of Infectious Diseases and Clinical Microbiology, Prof. Dr. Cemil Taşçıoğlu City Hospital, İstanbul, Turkey; 2 Department of Medical Microbiology, Faculty of Medicine, Acıbadem Mehmet Ali Aydınlar University, İstanbul, Turkey; 3 Department of Medical Biotechnology, Graduate School of Health Sciences,Acıbadem Mehmet Ali Aydınlar University, İstanbul, Turkey; 4 Department of Clinical Pharmacy, Faculty of Pharmacy, Altınbaş University, İstanbul, Turkey; 5 Department of Clinical Pharmacy, Graduate School of Health Sciences, İstanbul Medipol University, İstanbul, Turkey; 6 Department of Infectious Diseases and Clinical Microbiology, Faculty of Medicine, Acıbadem Mehmet Ali Aydınlar University, İstanbul, Turkey

**Keywords:** Antimicrobial agents, drug-resistant, multidrug-resistant, ceftazidime-avibactam combination, gram-negative bacteria

## Abstract

**Background/aim:**

Our study investigated the susceptibility rate of ceftazidime-avibactam and the risk factors associated with its resistance by analyzing gram-negative bacteria isolated from various patient samples.

**Materials and methods:**

Between March and November 2020, 1119 gram-negative bacteria strains were isolated from patient samples in Acıbadem Healthcare Group hospitals; ceftazidime-avibactam susceptibility results were evaluated using a 10/4μg (Oxoid, UK) disc and evaluated according to Eucast 2020 recommendations. Patient and isolate characteristics that could be risk factors were retrospectively investigated and statistically analyzed using SPSS 25.0.

**Results:**

Male patients made up 52% (n = 581) of the study’s total patient population, and they averaged 55.5 ± 24.9 years old. Of 1119 gram-negative strains culture and antibiogram, 1023 (91.4%) were sensitive to ceftazidime-avibactam. An increased risk of resistance was observed with female gender (OR = 2.29; CI 95% [1.45–3.61]; p < 0.05), *Pseudomonas aeruginosa* (OR = 1.67, CI 95% [1.03–2.7]; p < 0.05), the presence of multidrug-resistance (MDR) (OR = 4.07, CI 95% [2.47–6.7]; p < 0.05) pandrug-resistance (PDR) (OR = 12, (CI) 95% [9.9–14.7] ]; p < 0.05) and admission to intensive care unit (ICU) (OR = 1.89, CI 95% [1.22–2.93]; p < 0.05).

**Conclusion:**

The resistance rate of ceftazidime-avibactam was found to be 8.6%, and it was thought that resistant strains produced metallo-β-lactamase (MBL) type carbapenemase. Risk factors were female gender, *Pseudomonas aeruginosa*, MDR, PDR, and admission to ICU. Therefore, studying the ceftazidime-avibactam susceptibility test together with gram-negative bacteria identification, especially in groups at risk for resistance, is one of the important factors that can positively affect the success of treatment.

## 1. Introduction

Infections caused by antibiotic-resistant bacteria are one of the most serious public health challenges [1,2]. At the beginning of the 2000s, multidrug-resistant gram-positive bacteria were the most concerning; however, resistance gram-negative bacteria are now the center of attention [3]. These bacteria produce infectious diseases that are linked with considerable mortality and morbidity. Each year, more than 23,000 people in the United States die as a result of diseases caused by multidrug-resistant bacteria. When they researched these microbes, they discovered extended-spectrum beta-lactamase (ESBL) and carbapenemase producing *Enterobacteriaceae*, as well as multidrug-resistance (MDR) *Acinetobacter baumannii* and *Pseudomonas aeruginosa* [4]. Antibiotic resistance in gram-negative bacteria is mainly caused by enzymes that alter the structure of antibiotics, such as Amp C, cephalosporinase, ESBL, and carbapenemase [2]. It is well recognized that plasmid or transposon-mediated distribution promotes these resistance characteristics. The World Health Organization has warned that ESBL and carbapenemase-producing gram-negative bacteria, in particular, may cause intractable infectious disease and increased mortality [5]. Because the number of antibiotics available to treat carbapenem-resistant *Enterobacteriaceae* (CRE) is limited, it has become critical to seek for other antibiotic options. As a result of these advances, several novel antibiotic and combination treatment options have emerged in recent years [6]. One of these new combinations is the ceftazidime-avibactam combination. Avibactam is a nonbeta lactam, diazobicyclooctane-beta lactamase inhibitor. It is effective against metallo-beta-lactamases (MBL) from classes A, C, and D, but has minimal activity against MBL from class B. As a consequence, ceftazidime-avibactam has significant antimicrobial action against CRE and *P. aeruginosa*, but only moderate antimicrobial activity against *A. baumannii*, gram-positive bacteria, and anaerobic bacteria. Ceftazidime-avibactam combination is FDA-approved for treating severe urinary tract infections, intra-abdominal infections, ventilator-associated pneumonias, and healthcare-associated pneumonias [7]. In Turkey, Class D Oxacillinase (OXA) is the most common carbapenemase type, making ceftazidime-avibactam a potential therapy for carbapenem-resistant gram-negative infections [8,9]. However, studies show that resistance to ceftazidime-avibactam can develop with or without medication exposure [10,11]. Our goal was to gain a general understanding of ceftazidime-avibactam susceptibility at several hospitals in İstanbul, Turkey, and to identify risk factors associated with resistance.

## 2. Materials and methods

### 2.1. Study design

A total of 1119 gram-negative isolates obtained from several Acıbadem Healthcare Group hospitals in İstanbul, Turkey were retrospectively evaluated. All susceptibility tests were performed at Acıbadem Labmed Central Microbiology Laboratory with the Kirby-Bauer disc diffusion method, ceftazidime-avibactam 10/4 μg (Oxoid, UK) disc, and carbapenemase generation with 10 μg meropenem (BD BBL^TM^, USA) disc were utilized. All of the preceding steps incorporated Eucast 2020 recommendations. Patient demographic variables, including age, gender, sample type, infecting microorganisms, and admission department were collected. The resistance pattern of the strains was separated into two categories based on the antibiogram results: those with multiple antibiotic resistance and those without multiple resistance. Multiple resistance groups were identified in the study, including MDR (resistance to three or more antibiotic classes), XDR (maximally susceptible to two antibiotic classes), and PDR (resistance to all antibiotics).

### 2.2. Ethical approval and statistical analysis

This study was approved by the Acıbadem University Ethics Committee (date 14.10.2021/no 2021-20/18).

All the frequencies, including susceptibility and resistance to ceftazidime-avibactam, were provided as a percentage for each material type, microorganism type, ESBL and carbapenemase production, and drug resistance type. Crosstabs were used to show the relationship between variables such as age, department groups, and ceftazidime-avibactam susceptibility. The odds ratio for risk variables that impact ceftazidime-avibactam susceptibility and resistance was calculated using the chi-squared test. The SPSS 25.0 (Statistical Package for Social Sciences) program was used to conduct the statistical analysis.

## 3. Results

The demographic variables of the study include patients of both genders and belonging to numerous age groups. Among the patients 52% (n = 581) were male, 48% (n = 538) were female and the mean age was 55.5 ± 25.9 (min: 0 max: 97). 1023 (91.4%) strains are sensitive to ceftazidime-avibactam. When compared to other sample types, blood samples had the lowest sensitivity for ceftazidime-avibactam by 87.5%. 82% of the carbapenemase-producing gram-negative bacteria were sensitive to ceftazidime-avibactam and 7.2% of the resistant strains (60/837) were isolated from patients treated in clinical departments, whereas 12.8% (36/282) were from patients treated in intensive care units, (p < 0.05). Ceftazidime-avibactam resistant strains were found in 8.7% (43/496) of geriatric patients (≥ 65 years old) while 8.5% of (<65 years old) (53/623) nongeriatric patients, (p > 0.05). Table 1 shows the ceftazidime-avibactam sensitivity ratio sorted by sample types, while Table 2 shows the sensitivity ratio sorted by microorganism type and resistance pattern.

Females had an odds ratio of 2.29, while the presence of *P. aeruginosa*, MDR, PDR, and ICU admission has 1.67, 4.07, 12, and 1.89, respectively. The odds ratio of the factors that affect ceftazidime-avibactam is presented in Table 3.

## 4. Discussion

The ceftazidime-avibactam combination has received FDA approval in 2019 for the treatment of healthcare-associated ventilator-associated pneumonia, intra-abdominal infections and urinary tract infections caused by multi-drug resistant gram-negative bacilli [7]. This combination was granted a license in Turkey in 2019 [12], however it will not be included in the reimbursement health assurance system until April 28, 2021.

Prior to this date, ceftazidime-avibactam susceptibility rates of gram-negative bacteria isolated from various samples of patients followed in a private Acıbadem Healthcare Group hospitals between March-November 2020. At that time, the use of combination was limited to a few private health institutions. In general, resistance rises steadily as consumption rises [10,11]. As a result, we intended to investigate the susceptibility of this combination prior to its widespread use, in order to serve as a base for the future research.

The ceftazidime-avibactam resistant strains obtained from patient samples who are naive to this combination were studied in a multicenter study conducted in the United States between 2013 and 2016. Basal resistance was discovered to be primarily induced by intrinsic resistance generating MBL, and very rarely by porin mutation (OmpK36) [13].

In Turkey, molecular methods revealed that the dominant carbapenemase type is OXA-48, and MBL such as New Delhi metallo-beta-lactamase (NDM) and Verona integron-borne metallo-lactamase (VIM) are rarely found [14]. This situation leads us to believe that the combination of ceftazidime-avibactam may be an effective treatment for multidrug-resistant gram-negative bacterial infections in our country.

Another study from Turkey found that 95.2% of 84 enteric bacterial strains expressing OXA-*48* the most common kind of carbapenemase and *K. pneumoniae* carbapenemase (KPC) were susceptible to ceftazidime-avibactam [15]. The sensitivity of all gram-negative isolates to ceftazidime-avibactam was found to be 91.4% during our study duration. Using a liquid micro-dilution approach, ceftazidime-avibactam resistance was shown to be 20.1% in research including 167 *K. pneumonia* strains with carbapenem resistance [16]. In our study, 18% of gram-negative isolates with carbapenem resistance were resistant to ceftazidime-avibactam.

Another study, conducted in 2017 on 872 *K.pneumoniae* strains in China prior to the drug’s market entry, found that ceftazidime-avibactam resistance was only 3.7%. The resistant strains produced 53.1% MBL, 40.6% KPC type carbapenemase, and 6.3% produced both MBL and KPC [17]. Another study found that clinical success rates for ceftazidime-avibactam combinations ranged from 45% to 100% [18]. In our study, the sensitivity of gram-negative *Enterobacteriaceae* and *P. aeruginosa* isolates to ceftazidime-avibactam was determined to be 91% and 92% respectively. Another research reveals that using a combination of ceftazidime-avibactam for the treatment of *P. aeruginosa* resulted in an effective clinical response in 86.7% of the patients [19]. Similar to our study, a multicenter study conducted between 2016 and 2018 found that 92% of *P. aeruginosa* isolates were sensitive to ceftazidime-avibactam. The same study revealed that the rates of resistance varied greatly by region, ranging from 6% in Europe to 53.2% in South America [20]. A study reported that a total of 18.5% of 54 clinical *P. aeruginosa* isolates resistant to a variety of beta-lactam antibiotics were also resistant to ceftazidime-avibactam [21].

According to research conducted in Germany, 64.1% of multidrug-resistant/extensively drug-resistant (MDR/XDR) *P. aeruginosa* were responsive to ceftazidime-avibactam [22]. In our study, ceftazidime-avibactam sensitivity was found to be 95% for MDR bacteria and 78% for XDR bacteria. Additionally, we found that belonging to the MDR and XDR class bacteria group is considered a risk factor for ceftazidime-avibactam resistance. Furthermore, all four PDR isolates tested positive for ceftazidime-avibactam resistance.

In our study, the isolates were separated into groups based on the kind of sample and the patient’s admission to either intensive care unit (ICU) or clinical service. The susceptibility rates were lower in the blood sample and in samples taken from patients admitted to ICU. Furthermore, risk factor analysis showed that being admitted to the intensive care unit approximately double the risk for ceftazidime-avibactam resistance. A researchevaluated the antimicrobial susceptibility of gram-negative bacteria from intensive care unit and nonintensive care unit patients in United States hospitals and found that susceptibility rates of several antibiotics were usually lower in ICU patients compared to non-ICU patients [23].

Female gender, the isolated agent being *P. aeruginosa*, and the agent being from MDR or PDR resistance group were other risk factors for ceftazidime-avibactam resistance in our study while pneumonia and renal replacement were the risk factors for ceftazidime-avibactam resistance according to Ryan and his colleagues [24]. We could not make any judgments on renal function since our study did not include any clinical data about the patients’ kidney functions. From this standpoint and as a result, identifying patient groups at risk of developing resistance and conducting susceptibility tests for them is critical to avoiding treatment failure. The molecular analysis of carbapenemase types in the strains was not possible in our study because we used retrospective data from diagnostic routine tests and molecular analysis did not involve in this routine, which was a limitation of our study. Although this is one of our study limitations, future ceftazidime-avibactam susceptibility studies, in which molecular analysis can be used, will provide meaningful contributions to the literature. Our study, which included 1119 gram-negative bacteria strains isolated from patients who were followed up and treated in hospitals affiliated with a private health group, provides baseline resistance data in the population prior to the widespread use of ceftazidime-avibactam in Turkey. In our study this combination has high susceptibility rates compared to the available data from literature, indicating that it is a promising agent for the treatment of drug-resistant gram-negative bacteria. Antibiotic resistance increased dramatically with increased consumption [25], necessitating future research to evaluate the ceftazidime-avibactam sensitivity in the period following its frequent consumption in Turkey.

Ceftazidime-avibactam still has a high susceptibility rate, making it an excellent alternative for last-line therapy in the treatment of resistant gram-negative bacterial infections. However, resistance is evolving, which is considered a danger in females and in the presence of *P. aeruginosa*, MDR, PDR isolates, and patients admitted to ICU. More research is needed to determine the optimal approaches for avoiding future resistance based on our data.

## Figures and Tables

**Table 1:** Ceftazidime-avibactam sensitivity ratio according to sample types.

Sample types	% (n)	Ceftazidime-avibactam sensitivity% (n)
Urine	33.3% (n = 372)	93.2% (n = 347)
Trachea/aspirate culture	24.6% (n = 275)	90.5% (n = 49)
Pus	17.2% (n = 192)	89.5% (n = 172)
Blood	13%(n = 144)	87.5% (n = 126)
Biological fluid	4.5% (n = 50)	96.0% (n = 48)
Catheter	1.9% (n = 22)	95.4% (n = 21)
Biopsy material	1.5% (n = 17)	94.1% (n = 16)
Cerebrospinal fluid	0.2% (n = 3)	100% (n = 3)
Others	3.9% (n = 44)	93.1% (n = 41)
Total isolates	100% (n = 1119)	91.4% (n = 1023)

**Table 2:** Ceftazidime-avibactam sensitivity ratio according to microorganism type and resistance pattern.

Microorganism type/ resistance pattern	% (n)	Ceftazidime-avibactam sensitivity% (n)
Enterobacteria	65% (n = 728)	91% (n = 663)
*Klebsiella* spp.	61% (n = 680)	92% (n = 626)
Other enteric bacilli	4%(n = 48)	77% (n = 37)
*Pseudomonas* spp.	35% (n = 391)	92% (n = 359)
ESBL(+), Carbapenemase(−)	16% (n = 116)	99% (n = 115)
Carbapenemase(+)	47% (n = 345)	82% (n = 283)
Without multiple resistance	32.5% (n = 238)	100% (n = 238)
MDR	40% (n = 293)	95% (n = 278)
XDR	27% (n = 193)	78% (n = 148)
PDR	0.5% (n = 4)	

**Table 3:** Risk factors for development of Ceftazidime-avibactam resistance.

	Odds ratio	Lower limit	Upper limit	P-value
Gender Female/male	2.29	1.45	3.61	<0.001
*Pseudomonas aeruginosa* Present/absent	1.67	1.03	2.7	<0.05
MDR Present/absent	4.07	2.47	6.7	<0.05
PDR Present /absent	12	9.9	14.7	<0.05
Intensive care unit	1.89	1,22	2.93	<0.05
